# Interaction of Medicinal Plants and Their Active Constituents With Potassium Ion Channels: A Systematic Review

**DOI:** 10.3389/fphar.2022.831963

**Published:** 2022-02-22

**Authors:** Arezoo Rajabian, Fatemeh Rajabian, Fatemeh Babaei, Mohammadreza Mirzababaei, Marjan Nassiri-Asl, Hossein Hosseinzadeh

**Affiliations:** ^1^ Pharmacological Research Center of Medicinal Plants, Mashhad University of Medical Sciences, Mashhad, Iran; ^2^ Department of Pharmacodynamics and Toxicology, School of Pharmacy, Mashhad University of Medical Sciences, Mashhad, Iran; ^3^ Department of Clinical Biochemistry, School of Medicine, Shahid Beheshti University of Medical Sciences, Tehran, Iran; ^4^ Department of Clinical Biochemistry, School of Medicine, Kermanshah University of Medical Sciences, Kermanshah, Iran; ^5^ Department of Pharmacology, School of Medicine, Shahid Beheshti University of Medical Sciences, Tehran, Iran; ^6^ Neurobiology Research Center, Shahid Beheshti University of Medical Sciences, Tehran, Iran; ^7^ Pharmaceutical Research Center, Pharmaceutical Technology Institute, Mashhad University of Medical Sciences, Mashhad, Iran

**Keywords:** medicinal plants, phytochemicals, potassium channels, nociception, ischemia, diabetes

## Abstract

Potassium ion (K^+^) channels are pore-forming transmembrane proteins that control the transport of K^+^ ions. Medicinal plants are widely used as complementary therapies for several disorders. Studies have shown that the modulation of K^+^ channels is most likely involved in various pharmacological effects of medicinal plants. This review aimed to evaluate the modulatory effects of medicinal plants and their active constituents on K^+^ channels under pathological conditions. This systematic review was prepared according to the Preferred Reporting Items for the Systematic Reviews and Meta-analyses (PRISMA) 2020 guideline. Four databases, including PubMed, Web of Science, embase, and Scopus, were searched. We identified 687 studies from these databases, from which we selected 13 *in vivo* studies for the review by using the Population, Intervention, Comparison, Outcomes, Study (PICOS) tool. The results of the 13 selected studies showed a modulatory effect of medicinal plants or their active constituents on ATP-sensitive potassium channels (K_ATP_), and small (SK_Ca_) and large (BK_Ca_) conductance calcium-activated K^+^ channels in several pathological conditions such as nociception, brain ischemia, seizure, diabetes, gastric ulcer, myocardial ischemia-reperfusion, and hypertension via possible involvement of the nitric oxide/cyclic GMP pathway and protein kinase. K^+^ channels should be considered as significant therapeutic milestones in the treatment of several diseases. We believe that understanding the mechanism behind the interaction of medicinal plants with K^+^ channels can facilitate drug development for the treatment of various K^+^ channel-related disorders.

**GRAPHICAL ABSTRACT:**
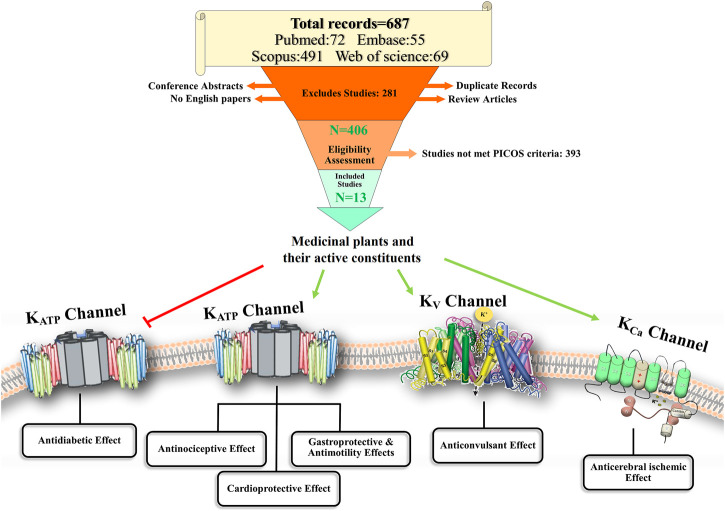


## Introduction

Potassium-selective ion channels are pore-forming proteins that allow the flow of potassium ions (K^+^) across the plasma membrane. K^+^ channels regulate a cell’s excitability and resting membrane potential and determine the shape of the action potential waveform in cells such as neurons and myocytes (Mathie et al., 2021). K^+^ channel families are classified into four groups: voltage-gated K (Kv), calcium-activated (K_Ca_), inwardly rectifying K (Kir), and two-pore domain potassium (K2P) channels (Taura et al., 2021).

Kv channels, the largest subset of K^+^ channels, assemble as homo- or hetero-tetramers, and the monomers form the central pore domain. Each monomer comprises six transmembrane segments (S1-S6) (Tian et al., 2014). They are activated by membrane depolarization and involved in many important physiological functions, including nervous and cardiac cellular excitability, regulation of hormone secretion such as the insulin release pathway, and immune response. Kv channels are mutated in some cardiac and nervous diseases, such as cardiac arrhythmias, epilepsy, episodic ataxia, and congenital deafness (Blunck and Batulan, 2012).

Calcium-activated K^+^ channels (K_Ca_) are formed by α-subunit tetramers (Kshatri et al., 2018). K_Ca_ channels have been categorized into three classes based on single-channel conductance: small conductance (SK_Ca_), intermediate conductance (IK_Ca_), and large conductance (BK_Ca_) calcium-activated K^+^ channels. There are eight members in this family of ion channels (Tian et al., 2014). K_Ca_ channels are expressed in a wide range of cells, including central nervous system cells, epithelial cells, blood cells, and arterial smooth muscle cells (Kshatri et al., 2018). These channels control the vascular tone, maintain K^+^ homeostasis, and regulate cellular excitability (Tano and Gollasch, 2014; Kshatri et al., 2018).

The Kir family consists of 15 members categorized into four functional groups. The most important subfamilies include the classical Kir channels (strong inward-rectifier K^+^ channel/Kir2.x), G-protein-activated Kir channels (GIRK, Kir3.x), ATP-sensitive K^+^ channels (K_ATP_, Kir6.x), and K^+^ transport channels (Kir1.x, Kir4.x, Kir5.x, and Kir7.x) (Tian et al., 2014). Kir channels are critical in the control of cellular excitability and K^+^ ion homeostasis (Mathie et al., 2021).

K2P channels have two pore domains per α-subunit, and each α-subunit contains four transmembrane (TM) segments (TM1-TM4) (Tian et al., 2014). K2P channels are considered “leak channels” for maintaining a negative membrane potential in various cells, including skeletal and heart myocytes, neurons, glia, and different types of epithelial cells (Enyedi and Czirják, 2010; Tian et al., 2014).

**FIGURE 1:**
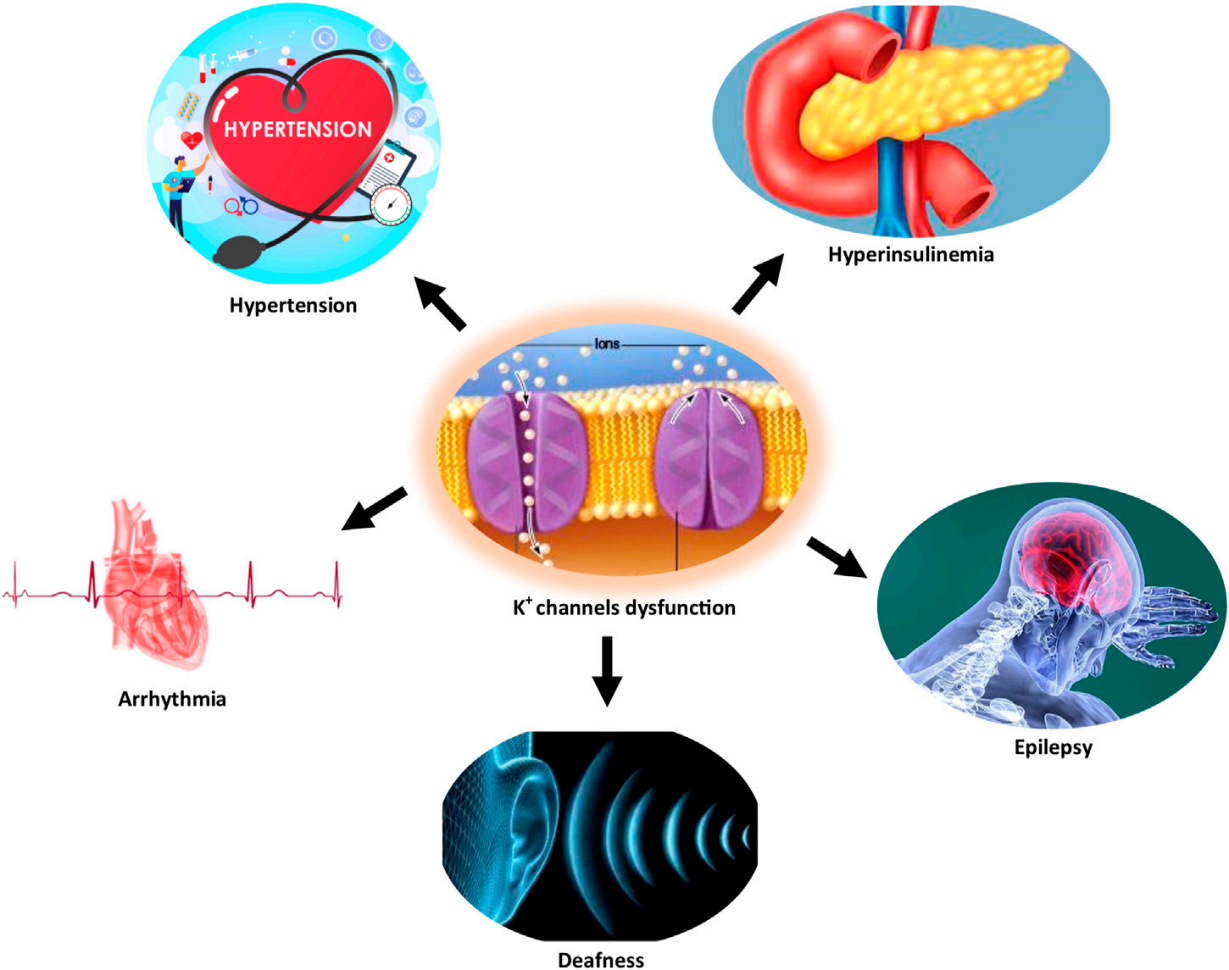
Some disorders are associated with K^+^ channels dysfunction.

Several studies have documented the pathophysiological role of the K^+^ channel in cardiac arrhythmia, hypertension, epilepsy, Alzheimer’s disease, type 2 diabetes mellitus, and age-related hearing loss (Tian et al., 2014; Burg and Attali, 2021; Singh et al., 2021). Figure 1 illustrates some disorders associated with K^+^ channel dysfunction.

Moreover, mitochondrial K_ATP_ channels are located in the intracellular membrane of mitochondria and composed of pore-forming (MITOK) and ATP-binding (MITOSUR) subunits. These channels play a vital role in mitochondrial physiology and are involved in the homeostatic control of cellular metabolism during stress conditions (Paggio et al., 2019).

For many years, studying K^+^ channels has been challenging due to the nonspecificity and other problems associated with classical pharmacological tools. However, several methods are currently available for studying K^+^ channels. Among them, manual voltage-clamp electrophysiology is considered the best method for measuring individual K^+^ channel activity. Other techniques include radioligand displacement, fluorescent sensors, and thallium flux assays (Weaver and Denton, 2021).

Given that clinically used drugs target only 7% of ligand-ion and 5% of voltage-gated channels, ion channels seem to be underrepresented in the drug discovery program (Djokic and Novakovic, 2020). This is because the chemical diversity information of ion channels available in databases is low, making drug designing against them all the more challenging (Bedoya et al., 2019). Moreover, another limitation is the lack of *in vivo* studies using specific ligands to determine the exact mechanism of interaction of herbal medicine or its active constituents with K^+^ channels. Although there are several *in vitro* studies on the interaction of herbal medicines with other channels or receptors, the lack of *in vivo* studies makes it difficult to differentiate the effects of herbal medicines on K^+^ channels from the other channels or receptors.

Several *in vitro* and *in vivo* studies have shown that herbal medicines and their active constituents have a variety of pharmacological properties, including antinociceptive, anxiolytic, antidepressant, antidiabetic, antiarrhythmic, antiischemic, gastroprotective, and vasorelaxant effects due to their selective targeting of K^+^ channels (Joseph et al., 2018; Zakaria et al., 2014; Zambrana et al., 2018; da Rosa et al., 2019; Imtiaz et al., 2019; Li et al., 2008; Li et al., 2019). Since medicinal plants and their active components are critical for modulating the K^+^ channels, in this review we focus on the interaction of medicinal plants and their constituents with the K^+^ channels. We believe this review will help identify the possible mechanisms of medicinal plants in various K^+^ channel-related disorders, which may further accelerate the drug design against these disorders.

## Methods

This systematic review was conducted as a guide for the Preferred Reporting Items for Systematic Reviews and Meta-Analyses (PRISMA) 2020 statement consisting of a 27-item checklist, the PRISMA abstract checklist, and a flow diagram (Page et al., 2021).

### Search Strategy

Search terms used in the four databases—Scopus, PubMed, Embase, and Web of Science—on April 27, 2021, were “Medicinal plants,” “Herbal medicine,” “Botany,” “Herb,” “Phytotherapy,” “Chinese Herbal Medicine,” “Herbal Preparations,” “Phytochemical,” “Ethnomedicine,’’ and “K^+^ channel’’ or “potassium channel.” The complete search strategy for all databases, including filters, is shown in Table 1. All titles and abstracts from each database were imported to the reference management software EndNote™ X9, in which duplicate references were excluded. Subsequently, all remaining studies were screened.

**TABLE 1:** Search terms used in Scopus, PubMed, Embase, and Web of science.

database	Search item
**Scopus**	TITLE-ABS-KEY (“potassium channels” OR “K channels”) AND (“medicinal plants” OR “herbal medicine” OR “herb” OR “phytochemicals” OR “Ethnomedicine” OR “Chinese Herbal Medicine” OR “phytotherapy”)
**PubMed**	(“potassium channels" [Title/Abstract] or “K channels" [Title/Abstract]) AND (“medicinal plants" [Title/Abstract] or “herbal medicine" [Title/Abstract] or “herb" [Title/Abstract] or “phytochemicals" [Title/Abstract] or “Ethnomedicine" [Title/Abstract] or “Chinese Herbal Medicine" [Title/Abstract] or “phytotherapy" [Title/Abstract])
**Embase**	(‘potassium channels':ti,ab, kw OR ′k channels':ti,ab,kw) AND (‘herbal medicine':ti,ab, kw OR ′medicinal plants':ti,ab, kw OR ‘ethnomedicine':ti,ab, kw OR ‘botany':ti,ab, kw OR ‘phytochemicals':ti,ab, kw OR ‘herb':ti,ab, kw OR ′chinese herbal medicine':ti,ab,kw) AND [1966–2021]/py
**Web of Science**	Title, abstract, keywords: (“potassium channels” OR K channels) AND (“herbal medicine” OR “medicinal plants” or “phytochemicals” OR “botany” OR “Chinese herbal medicine” OR “ethnomedicine")

### Inclusion/Exclusion Criteria

We designed a systematic search strategy by using the Population, Intervention, Comparison, Outcomes, and Study (PICOS) search tool (Methley et al., 2014; Bramer et al., 2018) and selected the articles that follow the PICOS design. The population included all animal models (male and female, of all ages). The intervention included the effect of medicinal plants or their active constituents on K^+^ channels under pathological conditions, including nociceptive, cerebral ischemia/reperfusion, seizure, diabetes, gastric ulcer, myocardial ischemia/reperfusion models, intestinal motility, and blood pressure. The comparison was between the control group and the group which was treated with the plant extract or its active components. The outcome was the effect of medicinal plants or their active constituents on nociception and writhing, cerebral ischemia, seizure, diabetes, gastric ulcer, intestinal motility, myocardial ischemia-reperfusion, and blood pressure. The study included all *in vivo* studies related to the effect of medicinal plants or their active constituents specifically on K^+^ channels and their possible mechanisms, except one study having both *in vivo* and *in vitro* experiments, from which only the *in vivo* experimental data was considered.

The exclusion criteria included review articles, books, editorials, conference abstracts, and letters; studies with no abstract or free full-text access; studies not written in English; studies that did not focus on the interaction of medicinal plants or their active constituents on K^+^ channels, *in vivo*; and studies on the role of K^+^ channels in plant physiology. Further, studies on mixed herbal components with a brand name or in combination with other drugs such as non-steroidal anti-inflammatory drugs, studies with unknown extraction methods, and *in silico*, *ex vivo*, and molecular docking studies were removed.

### Data Collection and Management

One author (MNA) evaluated the titles and abstracts of the electronic databases with the inclusion criteria. If a title and abstract met the inclusion criteria, the full text of that article was retrieved for further investigation. Two authors (MNA and FB) independently collected data from each full-text paper using the PICOS design and analyzed them. A third researcher (HH) confirmed the data from the study investigators. Data were stored in a file.

### Assessment Risk of Bias

The risk of bias (RoB) was assessed independently by two authors (MNA and FB), and disagreements were resolved by a third author (HH). The RoB tool was provided by the SYstematic Review Center for Laboratory animal Experimentation (SYRCLE) for animal intervention studies to assess the risk of bias, which contains 10 criteria (Hooijmans et al., 2014). This tool was adapted from the Cochrane Collaboration RoB tool used in clinical studies (Higgins et al., 2011). The RoB contains 1) sequence generation, 2) baseline characteristics, 3) allocation concealment, 4) random housing, 5) blinding caregivers and/or investigators, 6) random outcome assessment, 7) blinding outcome, 8) incomplete outcome data, 9) selective reporting of outcomes, and (10) other sources of bias (Hooijmans et al., 2014). These items were scored with ‘+’ low risk of bias, ‘−’ high risk of bias, and ‘?’ unclear risk of bias (Su et al., 2021).

## Results

### Selection of Articles

**FIGURE 2:**
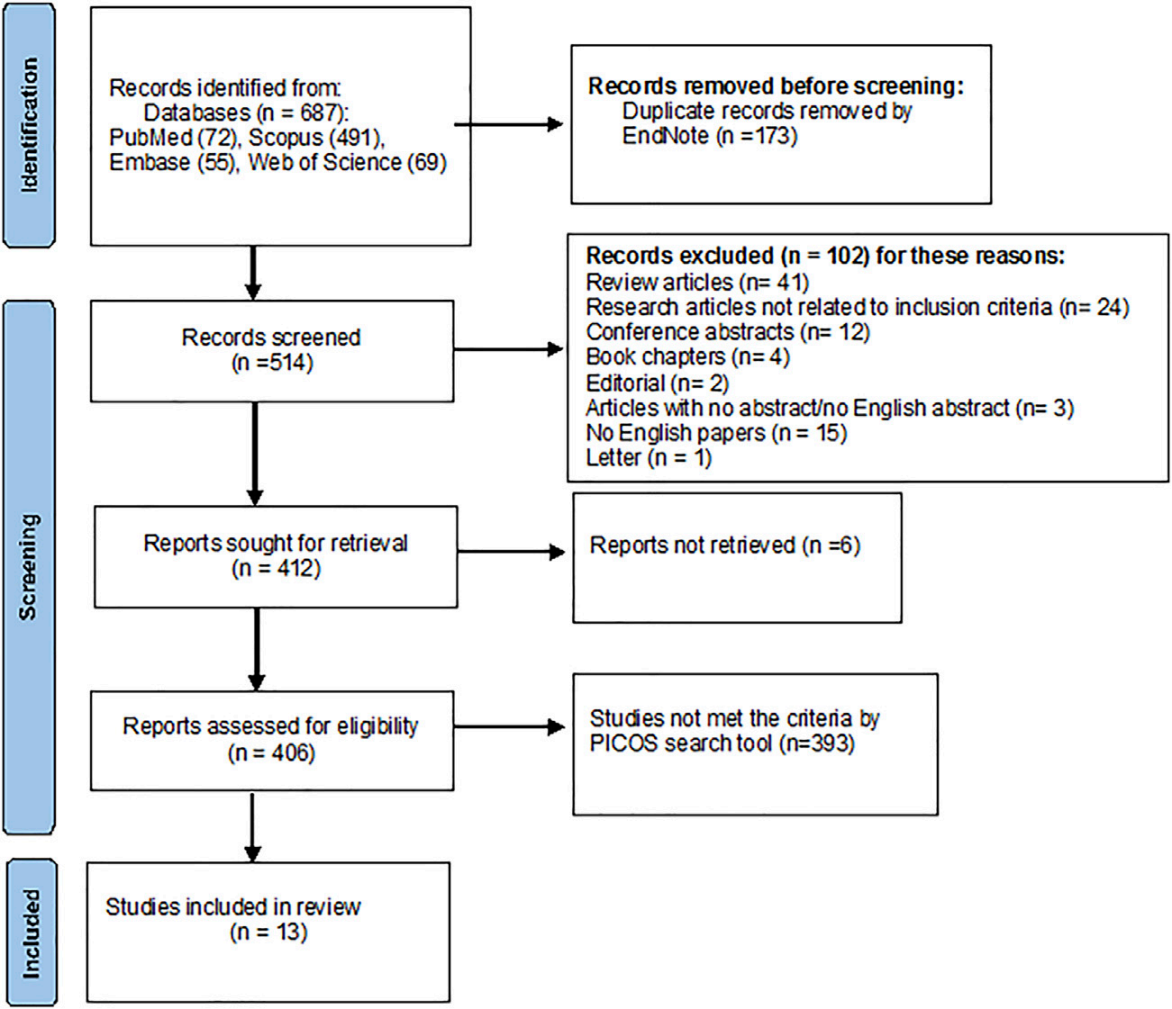
PRISMA flowchart diagram.

A total of 687 articles were identified from all the databases—PubMed (72), Scopus (491), Embase (55), and Web of Science (69). Duplicate records were removed using EndNote (*n* = 173). Subsequently, 514 articles were left. Following the inclusion criteria, another 102 records were excluded, as described in Figure 2. From the remaining 412 studies, 406 were assessed for eligibility, and 13 studies were included in the review. More details are shown in the PRISMA flowchart diagram in Figure 2.

### Characteristics of the Included Studies

In all the selected studies, the effects of medicinal plants and their active constituents on K^+^ channels have been described. A summary of the selected studies is shown in Table 2. There were six studies on the effect of medicinal plants or their active constituents on K_ATP_ channels in the peripheral nervous system. Adeyemi et al. (2018) reported that the hydroethanolic leaf extract of *Tetracera alnifolia* (HeTA; 50, 100, 200, and 400 mg/kg, p.o.) might have antinociceptive effects on acetic acid-induced writhing in mice. Pretreatment of animals with naloxone, l-arginine (L-Arg; precursor of nitric oxide (NO) synthase), or glibenclamide (a K_ATP_ channel inhibitor) prevented the antinociceptive effects; however, L-nitro-arginine could not reverse this effect. Hence, the antinociceptive effect of *T. alnifolia* may occur through the opioid/L-Arg-NO/K_ATP_ pathways (Adeyemi et al., 2018).

**TABLE 2:** The effects of medicinal plants and their active constituents on potassium channels.

N	Botanical drug (s)/Active constituents/Phytochemicals	Identified name/Family	Study design	Species/Strains/Gender	Number of animals	Experimental models	Assay	Dose (route)	Results	Main conclusion	References
1	Hydroethanolic leaf extract of *Tetracera alnifolia* (HeTA)	*Tetracera alnifolia* Willd/Dilleniaceae	*In vivo*	Mice/albino/male	6	Acetic acid-induced writhing (0.6% v/v, 10 ml/kg, i.p.)	Number of writhing (contraction of the abdominal musculature and extension of the hind limbs) alone or in the presence of naloxone, L-Arg, L-nitro-arginine, or glibenclamide	50, 100, 200, and 400 mg/kg, p.o	Reduced mean number of writhes	Antinociceptive property through opioid/L-Arg-NO/K_ATP_ pathways	Adeyemi et al. (2018)
2	Ethereal fraction from *Lecythis pisonis* leaves (LPEF)	*Lecythis pisonis* Cambess./Lecythidaceae)	*In vivo*	Mice/Swiss/male	6–11	Glutamate-evoked nociceptive response (20 µmol/paw)	Licking time alone or in the presence of naloxone, L-Arg, or glibenclamide	50 and 100 mg/kg, p.o	Reduced glutamate-induced nociception	Antinociceptive property through the opioid pathway, K^+^_ATP_. channels and negative modulation of L-Arg-NO	Brandão et al. (2013)
3	Methanol extract of the leaves of *Bougainvillea spectabilis* (MEBS)	*Bougainvillea spectabilis* Willd/Nyctaginaceae	*In vivo*	Mice/Swiss/male	5	Acetic acid-induced nociception (0.6% v/v, 10 ml/kg, i,p.)	Number of abdominal writhing, and percentages of pain inhibition alone or in the presence of methylene blue, or glibenclamide	MEBS (50, 100, or 200 mg/kg, p.o.	Reduced the number of writhing episodes and pain	Involvement of NO/cGMP/K_ATP_ pathways for antinociceptive effects	Ferdous et al. (2020)
4	Methanol extract of *Celosia cristata L.* (MECC)	*Celosia argentea* L./Amaranthaceae	*In vivo*	Mice/Swiss albino/male	5	Acetic acid-induced writhing (0.6% v/v, 10 ml/kg, i.p.)	Number of writhing alone or in the presence of methylene blue, or glibenclamide	50,100,200,400 mg/kg, p.o	Reduced the number of writhing	Association between the antinociceptive activity with cGMP pathway, and K_ATP_^+^ channel	Islam et al. (2016)
5	Essential oil of *Zingiber zerumbet* (EOZZ)	*Zingiber zerumbet* (L.) Roscoe ex Sm/Zingiberaceae	*In vivo*	Mice/ICR/male	10	Acetic acid-induced abdominal writhing test (0.6% v/v, 10 ml/kg, i.p.)	Number of writhing alone or in the presence of L-Arg, methylene blue, or glibenclamide	50, 100, 200, 300 mg/kg, i.p	Reduced the number of writhing, and increase the percent of inhibition	The participation of L- Arg/NO/cGMP/K_ATP_ pathway for antinociceptive activity	Khalid et al., 2011
6	3,3′,5,6,7,8-hexamothoxy-4′,5′-methylenedioxyflavone, 3,3′,4′,5′,5,6,7,. 8-octamethoxyflavone (exoticin), 6,7,4′,5′-dimethylenedioxy-3,5,3′-trimethoxyflavone, and 3,3′,4′,5,5′,8-hexamethoxy-6,7-methylenedioxyflavone,. active constituents of methanol extract of *N. plumbaginifolia* leaves	*Nicotiana plumbaginifolia* Viv./Solanaceae	*In vivo*	Mice/Swiss albino/male	6	Acetic acid-induced writhing test (1% w/v, 10 ml/kg, i.p.)	The onset of writhing, and the number of writing episodes alone or in the presence of glibenclamide	12.5, 25 mg/kg, p.o	Increased writhing onset time and decreased the writhing episodes	Involvement of K_ATP_ channel for antinociceptive effect	Shajib et al. (2018)
7	Total flavone of *Rhododendron* (TFR)	*Rhododendron simsii* Planch/Ericaceae	*In vivo*	Rats/Sprague-Dawley/male	4	Cerebral brain ischemia/reperfusion model (Ischemia for 20 min followed by 2 h reperfusion)	1) Morphological changes (Nissl staining) alone or in the presence of apamin, TRAM-34, or HC-067047. 2) Protein expression (Western blot) alone or in the presence of apamin, TRAM-34, or HC-067047. 3) The Ca^2+^ fluorescence intensity (Laser scanning confocal experiment) alone or in the presence of apamin, TRAM-34, or HC-067047	100 mg/kg, i.v	1) Improved the pathological injury of the cerebral cortex. 2) Increased protein expression of SK_Ca_, IK_Ca_, and TRPV4 channels in the endothelial cells from CBA. 3) Reduced the mean fluorescence intensity of Ca^2^ in the smooth muscle cells of CBA	The involvement of BK_Ca_ channels for anticerebral ischemia-reperfusion injury	Han et al. (2018)
8	*Pseudospondias microcarpa* (A. Rich) Engl. hydroethanolic leaf extract (PME)	*Pseudospondias microcarpa* (A.Rich.) Engl./Anacardiaceae	*In vivo*	Male/ICR/mice	10	4-AP-induced seizures (12 mg/kg, i.p.)	Latencies for the onset of convulsive episodes (clonic or tonic), and death. Clonic seizures (appearance of facial myoclonus, forepaw myoclonus, and forelimb clonus), tonic seizures (explosive clonic seizures. with wild running and tonic forelimb and hind limb extension) alone or in the presence of 4-AP	30, 100 or 300 mg/kg, p.o	Delayed the latency of both clonic and tonic seizures. Protected against clonic and tonic seizures	The involvement of activation of K^+^ channel in anticonvulsant effects	Adongo et al. (2017)
9	*Belamcanda chinensis* water leaf extract (BCL)	*Belamcanda chinensis* (L.) DC./Iridaceae. Synonym of *Iris domestica* (L.) Goldblatt & Mabb/Iridaceae	*In vivo*	Rats/Wistar/male	6	1) Normal rats. 2) STZ-induced diabetic rats (50 mg/kg, i.p.)	1) Fasting blood glucose, serum insulin levels alone or in the presence of nicorandil or nifedipine. 2) Oral glucose tolerance	400, 800, 1600 mg/kg, p.o	1) Lowered fasting blood glucose levels, oral glucose tolerance, and increased serum insulin concentration in normal rats. 2) Lowered fasting blood glucose levels and improved oral glucose tolerance in diabetic rats	The involvement of closing K_ATP_ and opening Ca^2+^ channels for antidiabetic effect	Wu et al. (2011)
10	Hydroethanolic extract of *Cochlospermum regium* (Mart. ex Schrank) Pilg. (HECr)	*Cochlospermum regium* (Schrank) Pilg./Bixaceae	*In vivo*	Mice/Swiss/female	6	Ethanol-induced gastric ulcer (0.3 M HCl/70% ethanol, p.o.)	Measured ulcerated area by a percentage of the total area of the gastric stomach (mm^2^) alone or in the presence of indomethacin, l-NAME, glibenclamide, or yohimbine	25, 100, 400 mg/kg, p.o	Reduced percent of the ulcered area	The gastroprotective effect through non-specific complexes, including activation of K_ATP_ channels, α_2_-adrenergic receptors, and stimulation of PGs and NO	Arunachalam et al. (2019)
11	Ethanol extract of *Maytenus Erythroxylon* (ME)	*Maytenus erythroxylon* Reissek/Celastraceae	*In vivo*	Mice/Swiss/male	7	Alterations in normal intestinal transit, a model that induced after 60 min of the pretreatment (10 ml/kg, p.o.) black marker (5% charcoal suspension in 5% Arabic gum)	Measured percent of intestinal transit = Length traveled by charcoal meal/Total intestinal length × 100 alone or in the presence of glibenclamide, l-NAME, or propranolol	62.5, 125, 250 and 500 mg/kg, p.o.	Reduced the percentage of intestinal transit	Involvement of the NO/cGMP/K_ATP_ pathway, and tissue adrenergic receptors modulation for antimotility	Formiga et al. (2017)
12	Polydatin	-	*In vivo*	Rats/Sprague Dawley/male	10	Myocardial ischemia/reperfusion	1) Monitored heart rate via subcutaneous stainless-steel electrodes alone or in the presence of 5HD, chelerythrine, or GF 2) Measures area at risk, CPK, and LDH	20 μg/kg, IV	1) Reduced heart rate, and infarct size 2) Decreased the release of CPK and LDH from the damaged myocardium	The involvement of PKC-K_ATP_ dependent signaling for antiischemic/reperfusion injury	Miao et al. (2011)
13	Ethanol soluble fraction from *Acanthospermum hispidum* (ESAH)	*Acanthospermum hispidum* DC./Compositae	*In vivo*	Rats/Wistar/male	5	Normotensive rats	Monitored mean arterial pressure, and systolic blood pressure by left carotid artery that was cannulated and connected to a pressure transducer alone or in the presence of l-NAME, methylene blue, or TEA	30, 100, 300 mg/kg, intraduodenal	Induced acute hypotensive effect	The involvement of the NO/cGMP/K^+^ channels in the hypotensive response	Tirloni et al. (2017)

Brandão et al. (2013) investigated the antinociceptive effect of the ethereal fraction of *Lecythis pisonis* leaves (LPEF; 50 and 100 mg/kg, p.o.) on the glutamate-evoked nociceptive response in mice. LPEF reduced nociception, and pretreatment with naloxone, L-Arg, or glibenclamide antagonized this effect. Hence, it seems that LPEF exerts its antinociception effect via the opioid/K_ATP_/L-Arg-NO pathways (Brandão et al., 2013).

Ferdous et al. (2020) investigated the antinociceptive effects of the methanol extract of *Bougainvillea spectabilis* leaves (MEBS; 50, 100, and 200 mg/kg, p.o.) on acetic acid-induced writhing in mice. MEBS reduced the number of writhing episodes and pain. Pretreatment of animals with methylene blue (as an inhibitor of the cGMP pathway) synergized the antinociceptive effect of MEBS; in contrast, pretreatment with glibenclamide reversed the antinociceptive effect. These results suggest that MEBS have antinociceptive effects, possibly through the modulation of K_ATP_ channels and cGMP (Ferdous et al., 2020).

Islam et al. (2016) found that the methanol extract of *Celosia cristata* (MECC; 50, 100, 200, and 400 mg/kg, p.o.) has antinociceptive effects on the acetic acid-induced writhing in mice. For the mechanistic evaluation of the antinociceptive activity of MECC, they pretreated the animals with various compounds. Pretreatment with glibenclamide reversed the antinociceptive effect of MECC, whereas co-administration of methylene blue with MECC (400 mg/kg) amplified the antinociceptive activity. These results indicate that the antinociceptive effect of MECC may be partly related to the cGMP and K_ATP_ channels. However, the antinociceptive effects of MECC on central and peripheral nervous systems have been shown in several nociception tests including formalin and glutamate-induced paw licking and edema, immersion test, and hot plate test. From these tests, the possible role of the opioid system was indicated. However, in this review, we considered only the part of the study that evaluated the role of K^+^ channels in antinociception (Islam et al., 2016).

Khalid et al. (2011) found that the essential oil of *Zingiber zerumbet* (EOZZ; 50, 100, 200, and 300 mg/kg, i.p.) has an antinociceptive effect in acetic acid-induced writhing in mice. The initial results showed that the i.p. route of EOZZ administration was more potent than the p.o. route. Pretreatment of animals with L-Arg and glibenclamide reversed the antinociceptive effect of EOZZ (200 mg/kg), while pretreatment with methylene blue enhanced the antinociceptive activity. It seems that EOZZ acts via the K_ATP_ channels and modulates the L-Arg/NO/cGMP pathway, apart from its possible involvement in the inhibition of the glutamatergic system and transient receptor potential vanilloid 1 (TRPV4) receptors (Khalid et al., 2011).

Shajib et al. (2018) reported that polymethoxyflavones (PMFs; four compounds), the active constituents of the methanol extract of *Nicotiana. plumbaginifolia* leaves (12.5 and 25 mg/kg, p.o.), have antinociceptive effects in writhing tests in mice. However, pretreatment of animals using glibenclamide decreased the protective effects of these PMFs. For more details, see Table 2. Moreover, the antinociceptive effect of 6,7,4′,5′-dimethylenedioxy-3,5,3′-trimethoxyflavone was greater than that of the other PMFs. Opioid receptors have also been implied in the antinociceptive effect of these PMFs. Hence, these results suggest that the antinociceptive effect of PMFs may be related to the ATP-sensitive K^+^ channel opening and the opioid system, apart from the possible suppression of inflammatory mediators such as prostaglandins (PGs), cyclooxygenase, lipoxygenase (Shajib et al., 2018).

Han et al. (2018) investigated the role of the total flavon of *Rhododendron* (TFR) on K^+^ channels in cerebral brain ischemia/reperfusion in rats. TFR (100 mg/kg, i.v.) improved the pathological injury of the cerebral cortex. Moreover, it increased the protein expression of TRPV4, IK_Ca_, and SK_Ca_ channels in the endothelial cells from the cerebral basal artery (CBA), as well as reduced the mean fluorescence intensity of Ca^2+^ in the smooth muscle cells of CBA. These results suggest that the activation of the TRPV4-dependent pathway has two consequences. First, it opens the endothelial IK_Ca_/SK_Ca_ channels, which in turn leads to the hyperpolarization of the endothelium and smooth muscle cell membranes. Second, it activates the BK_Ca_ channel and reduces Ca^2+^ in smooth muscle cells of CBA (Han et al., 2018).

The study by Adongo et al. (2017) investigated the effects of *Pseudospondias microcarpa* hydroethanolic leaf extract (PME) on the activation of K^+^ channels in the 4-aminopyridine (4-AP)-induced seizures in mice. They reported that PME (30, 100, 300 mg/kg, p.o.) delayed the latency of both clonic and tonic seizures and protected against 4-AP-induced seizures. It seems that PME acts via the direct activation of the K^+^ channel and membrane hyperpolarization or through the inhibition of the glutamate signaling pathway. However, this study also found the possible involvement of other systems in different seizure models in animals (Adongo et al., 2017).

Wu et al. (2011) investigated the role of *Belamcanda chinensis* leaf extract (BCL) on the K_ATP_ channel in normal and streptozotocin (STZ)-induced diabetic rats. BCL (400, 800, 1,600 mg/kg, p.o.) lowered the fasting blood glucose levels and oral glucose tolerance in both normal and diabetic animals. Moreover, it increased serum insulin levels in normal rats. However, this effect was reversed in the presence of nicorandil (an ATP-sensitive K^+^ ion channel opener) and nifedipine (a Ca^+2^ ion channel blocker). These results indicate that BCL lowers glucose levels and stimulates insulin secretion by closing K^+^_ATP_ and opening Ca^2+^ channels (Wu et al., 2011).

Arunachalam et al. (2019) investigated the effect of the hydroethanolic extract of *Cochlospermum regium xylopodium* (HECr) on the K^+^_ATP_ channel in an acute gastric ulcer mouse model. They demonstrated the gastroprotective effect of HECr (25, 100, 400 mg/kg, p.o.) in acidified ethanol-induced gastric ulcers in mice. Pretreatment of animals with glibenclamide reduced the antiulcer activity of HECr (100 mg/kg). Furthermore, pretreatment with indomethacin (an inhibitor of PGs), N-nitro-l-arginine methyl ester (l-NAME, a non-selective nitric oxide synthase inhibitor), or yohimbine (α_2_-adrenoreceptor antagonist) reversed the gastroprotective effect of HECr. Hence, HECr seems to have a gastroprotective effect non-specifically through the activation of K^+^_ATP_ channels and α_2_-adrenergic receptors, and the stimulation of PGs and NO (Arunachalam et al., 2019).

Formiga et al. (2017) investigated the role of *Maytenus erythroxylon* (Me) extract on intestinal motility and the possible involvement of the K^+^_ATP_ channel in mice. Me (62.5, 125, 250, and 500 mg/kg, p.o.) reduced the percentage of intestinal transit in mice, which was reversed by the pretreatment of animals with glibenclamide, l-NAME, or propranolol. These results indicate that the effect of ME extract on intestinal motility may involve the NO/cGMP/K_ATP_ pathways, apart from the modulation of adrenergic receptors (Formiga et al., 2017).

We also found two studies related to the effects of medicinal plants and phytochemicals on the cardiovascular system. Miao et al. (2011) showed that polydatin, a stilbene compound, has a cardioprotective effect in a rat model of myocardial ischemia/reperfusion. Polydatin reduced the heart rate and infarct size, whereas this effect was reversed by 5-hydroxydecanoate (5-HD), a selective blocker of mitochondrial K_ATP_ channels, and two potent protein kinase C (PKC) inhibitors, chelerythrine or GF109203X (GF). In addition, the decrease in the release of creatine phosphokinase (CPK) and lactate dehydrogenase (LDH) by polydatin was abolished in the presence of 5-HD, chelerythrine, or GF. These results suggest that the cardioprotective effects of polydatin may be related to the activation of PKC-K^+^_ATP_ signaling, apart from its free radical scavenging activity that was indicated as a different mechanism for the cardioprotective effect (Miao et al., 2011).

Tirloni et al. (2017) showed that ethanol-soluble fractions from *Acanthospermum hispidum* (ESAH; 30, 100, and 300 mg/kg, intraduodenal) has a hypotensive effect in normotensive rats, and pretreatment of animals with l-NAME, methylene blue, or tetraethylammonium (TEA; a nonspecific K^+^ channel blocker) prevented this effect. These results suggest the involvement of the NO/cGMP/K^+^ channels in the hypotensive response of ESAH (Tirloni et al., 2017).

## Discussion

Of the 13 studies discussed in this systematic review, six examined the antinociceptive effects of medicinal plants and their possible involvement with the K^+^_ATP_ channel. Two studies investigated the neuroprotective effect of medicinal plants and their modulation of the SK_Ca_ and IK_Ca_ channels. One study examined the anti-diabetic effect of a medicinal plant that acts by closing the K^+^_ATP_ channel. Two studies evaluated the therapeutic effects of medicinal plants on gastric ulcers and intestinal motility, possibly via modulating the K^+^_ATP_ channels. Finally, two studies examined the cardioprotective effect of medicinal plants through the possible involvement of K^+^ channels.

In all 13 studies, K^+^ channels were demonstrated as a possible pharmacological target of medicinal plants or their active constituents. Hence, in this systematic review, we will focus on the molecular mechanism of the interaction between medicinal plants and their active constituents with K^+^ channels to evaluate if it can be a novel drug target in the treatment of various diseases.

### The Modulatory Effects of Medicinal Plants or Their Active Constituents on K^+^ Channels in the Nervous System

#### The Involvement of NO/cGMP/K_ATP_ Pathway for Antinociceptive Effects

The antinociceptive effects of medicinal plants HeTA, LPEF, MEBS, MECC, EOZZ, and polymethoxyflavones (active constituents of the methanol extract of *N. plumbaginifolia* leaves) are linked to the modulation of K_ATP_ channels in six studies (Khalid et al., 2011; Brandão et al., 2013; Islam et al., 2016; Adeyemi et al., 2018; Shajib et al., 2018; Ferdous et al., 2020). K_ATP_ channels are widely distributed in both the central and peripheral nervous systems. These channels have several physiological functions, including the regulation of neuronal excitability and suppression of hyperalgesia (Li et al., 2021).

Kir6.2, SUR1, and SUR2 are expressed in the dorsal root of the ganglia (DRG) and are important for inhibiting hyperalgesia during severing neurons (Zoga et al., 2010; Tinker et al., 2018). It has been shown that intracellular calcium [Ca^2+^]_i_ 57 or 610 nM activates DRG neuronal K_ATP_ channels via the Ca^2+^/Ca^2+^-calmodulin/CaM-dependent kinase II (Ca^2+^/CaM/CaMKII) signaling pathway. This in turn opens K^+^ channels, reduces excitability, and exerts possible antihyperalgesic effects. It is suggested that the opening of K_ATP_ channels can serve as a novel analgesic target in the treatment of neuropathic pain (Kawano et al., 2009b). NO activated K_ATP_ channels in large DRG neurons in the SUR1 subunit through direct S-nitrosylation of cysteine residues (Kawano et al., 2009a).

Furthermore, the effect of medicinal plants or phytochemicals on NO/cGMP signaling was demonstrated in five studies (Khalid et al., 2011; Brandão et al., 2013; Islam et al., 2016; Adeyemi et al., 2018; Ferdous et al., 2020). The studies indicated that the antinociceptive effects of medicinal plants or phytochemicals depend on the activation of ATP-dependent K^+^ channels due to the modulation of the NO/cGMP signaling pathway.

However, NO has diverse roles in the modulation of analgesia. It may have a nociceptive or antinociceptive effect depending on the animal model, time, dose, and route of administration (Sousa and Prado, 2001; Cury et al., 2011; Staurengo-Ferrari et al., 2014). Several studies have shown the involvement of the l-arginine/NO/cGMP/K_ATP_ channel pathway in antinociceptive action (Ghorbanzadeh et al., 2019; Alizamani et al., 2021).

**FIGURE 3:**
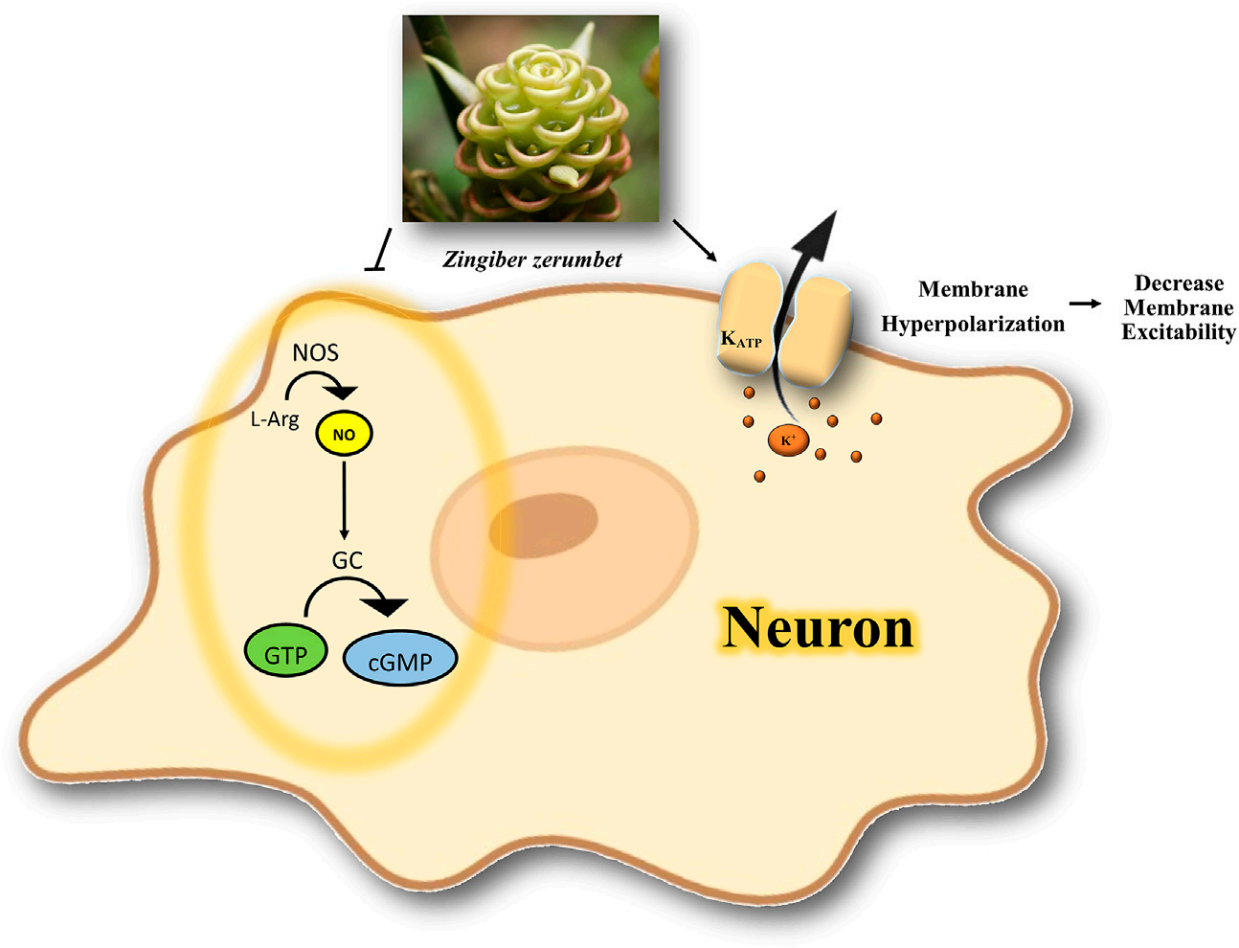
Possible mechanism of the interaction of medicinal plants or phytochemicals with K^+^ channels in nociception. The activation of ATP-dependent K^+^ channels and modulation of NO/cGMP signaling pathway is involved in antinociception. NOS, nitric oxide synthase; NO, nitric oxide; L-Arg, l-Arginine; GC, guanylate cyclase; GTP, guanosine Triphosphate; cGMP, cyclic guanosine monophosphate; K^+^, potassium; K_ATP_, ATP-sensitive potassium channel.

In this review, based on inclusion criteria, we only checked whether cGMP or NO inhibitors could boost the antinociceptive effect of medicinal plants or their active constituents; these medicinal plants or phytochemicals act via modulating the NO/cGMP/K_ATP_ channel pathway. Further studies on their role in pain treatment are needed to evaluate their therapeutic potential by targeting the NO/cGMP/K_ATP_ channel pathway. A possible mechanism of the interaction of medicinal plants or phytochemicals with K^+^ channels in nociception is illustrated in Figure 3.

#### The Involvement of BK_Ca_ Channels for Anticerebral Ischemia-Reperfusion Injury

**FIGURE 4:**
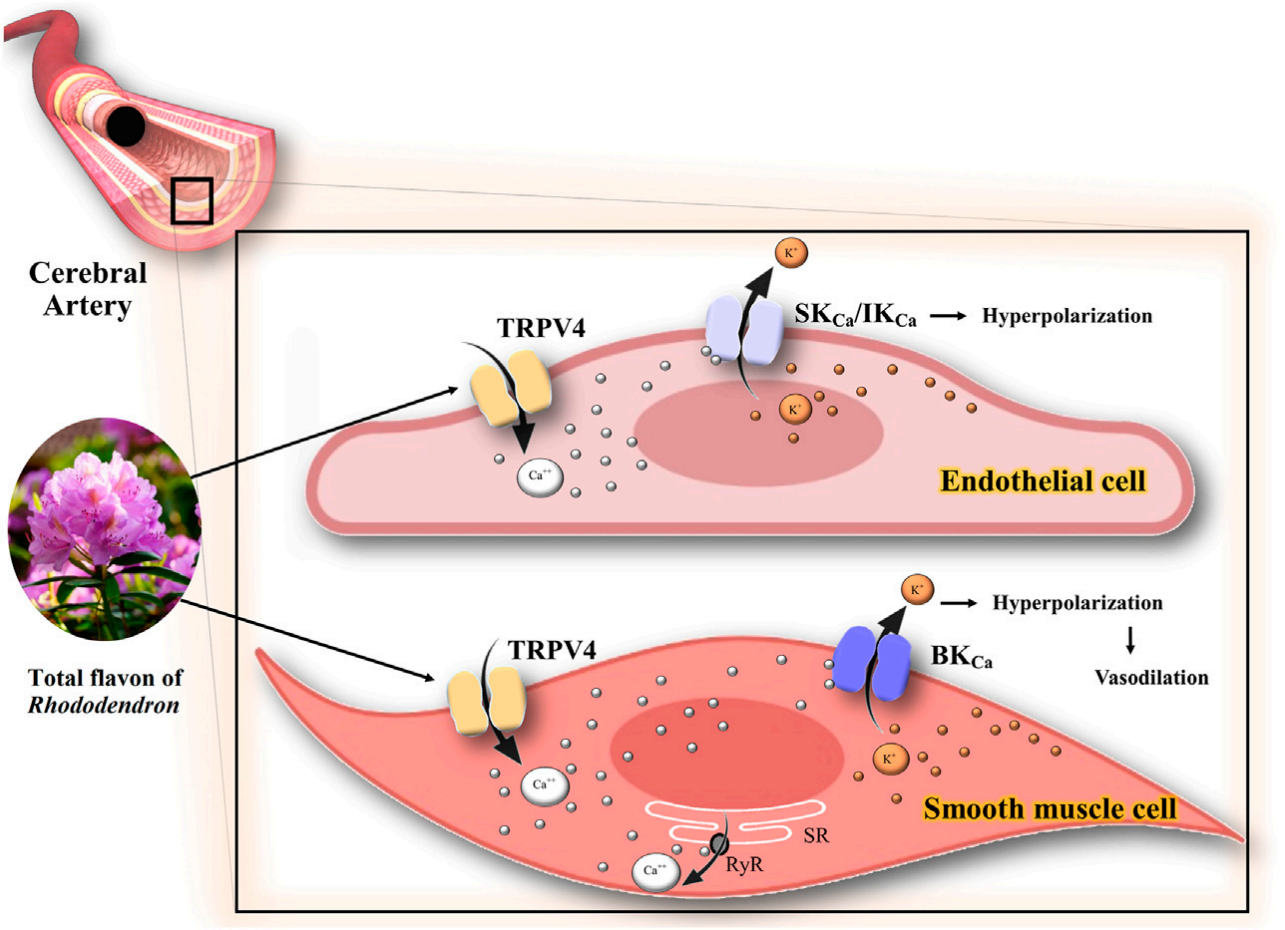
Possible mechanism of the interaction of TFR with K^+^ channels in ischemia. The activation of TRPV4 channels has two consequences: 1) Opening of the endothelial IK_Ca_ IK_Ca_/SK_Ca_ channels, which in turn leads to the hyperpolarization of the endothelium and smooth muscle cell membranes. 2) Stimulation of Ca^++^ release from ryanodine receptors in the sarcoplasmic reticulum and opening the BK_Ca_ channels, thereby leading to hyperpolarization and vasodilation in smooth muscle cells of CBA. TRPV4, transient receptor potential vanilloid 4; Ca^++^, calcium; K^+^, potassium; IK_Ca_, intermediate conductance calcium-activated K^+^ channels; SK_Ca_, small conductance calcium-activated K^+^ channels; BK_Ca_, large conductance calcium-activated K^+^ channels; RyR, ryanodine receptors; SR, sarcoplasmic reticulum.

Han et al. (2018) demonstrated the protective effect of TFR on ischemic brain injury and determined the functions of TRPV4, SK_Ca_, IK_Ca,_ and BK_Ca_ channels in cerebral ischemia-reperfusion (Han et al., 2018). There is a link between the activation of TRPV4 and the opening of BK_Ca_ channels in the smooth muscle cells that cause dilation in the rat cerebral arteries, resulting in the improvement of hypoperfusion in the infarcted area (Liu et al., 2020b). This vasodilation occurs by the entry of Ca^2+^ through TRPV4 channels that stimulate Ca^2+^ release from ryanodine receptors in the sarcoplasmic reticulum. Subsequently, Ca^2+^ sparks are induced, activating the BK_Ca_ channels, thereby leading to hyperpolarization and vasodilation (Liu et al., 2020a). The possible mechanism of the interaction of medicinal plants or phytochemicals with K^+^ channels in ischemia is summarized in Figure 4. The neuroprotective effects of BK_Ca_ channels in cerebral ischemic stroke have been reported (Liu et al., 2020b). Thus, studying the role of TFR or a similar compound on BK_Ca_ channels could lead to the development of a potential therapeutic target in ischemia-reperfusion injury.

#### The Involvement of K^+^ Channel in Anticonvulsant Effects

PME could have an anticonvulsant effect in the 4-AP-induced seizure. 4-AP is an antagonist of Kv channels (Adongo et al., 2017). It can be administered systemically or intracerebrally to animals to study the anticonvulsant activity of drugs. 4-AP is also a stimulator of voltage-gated Ca^+2^ channels and contributes to the release of excitatory neurotransmitters such as glutamate (Brito et al., 2009). Recently, the 4-AP model has been used to detect antiepileptic effects in new-generation drugs and induce seizure-like events in *in vitro* studies (Heuzeroth et al., 2019). Therefore, the activation of K^+^ channels by medicinal plants like PME can be an important drug target in the treatment of seizures. Further studies are needed on the anticonvulsant activity of PME to investigate the type of Kv channel involved in the protection against 4-AP.

### The Involvement of K_ATP_ Channels for Antidiabetic Effect

**FIGURE 5:**
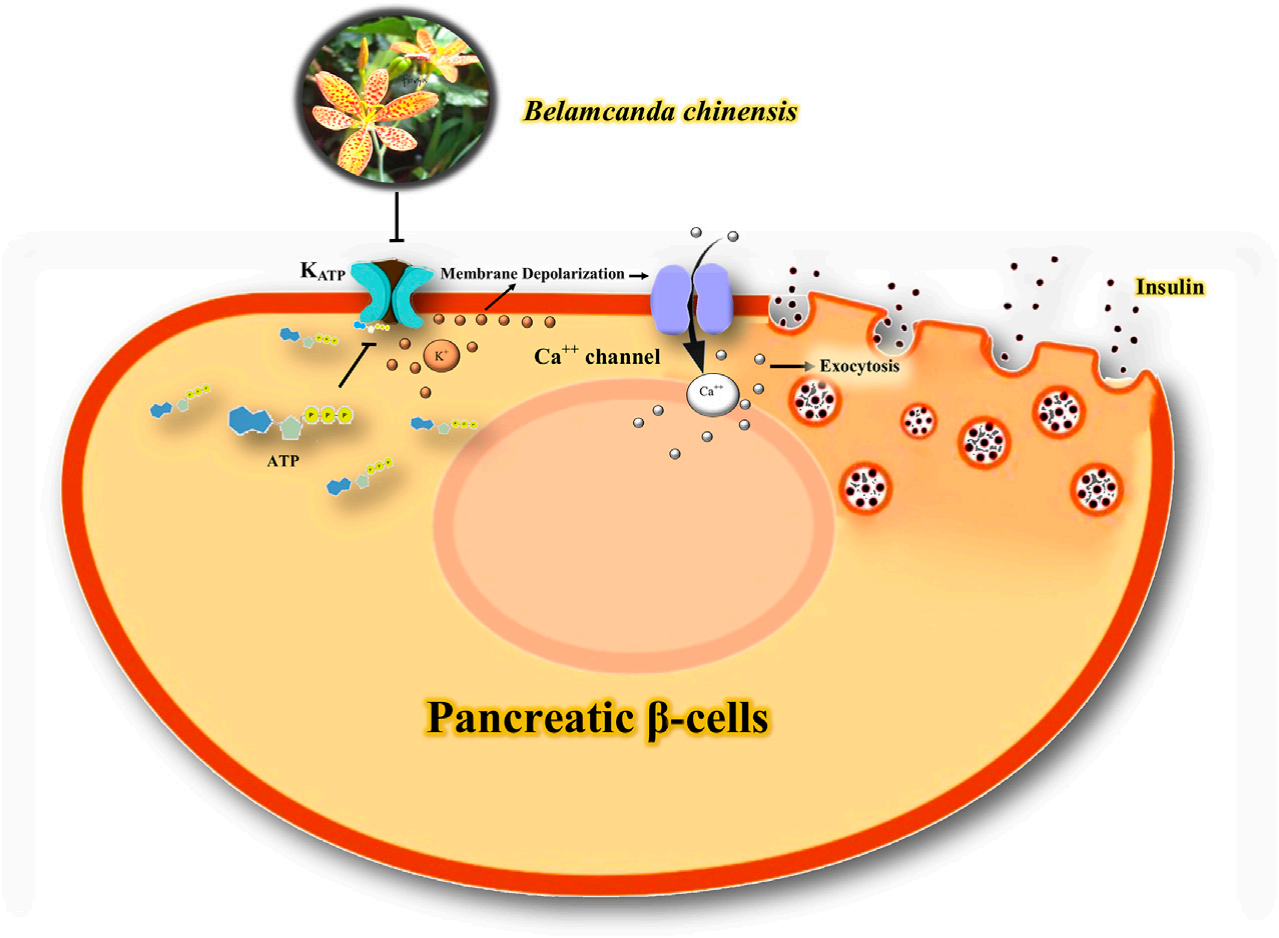
Possible mechanism of the interaction of BCL with K^+^ channels in diabetes. Blockage of K_ATP_ channels in the cell membrane leads to depolarization of the membrane and an increase in Ca^2+^ influx, resulting in exocytosis of insulin granules. ATP, adenosine Triphosphate; K_ATP_, ATP-sensitive potassium channel; Ca^++^, calcium; K^+^, potassium.

The study by Wu et al. (2011) indicates that BCL lowers glucose levels and increases insulin secretion by closing K_ATP_ channels and opening Ca^2+^ channels. Isoflavone glycosides may be involved in the antidiabetic effect of BCL (Wu et al., 2011). K_ATP_ channels are critical in the release of insulin from pancreatic β cells (Tinker et al., 2018). The combination of the subunits of Kir6.2/SUR1 of the K_ATP_ channel in pancreatic β-cells regulates the release of insulin (Li et al., 2021). K_ATP_ channels provide a link between adenine nucleotides and electrical activity following changes in blood glucose levels in β cells (Rustenbeck et al., 2021). High glucose levels block K_ATP_ channels in the cell membrane, leading to depolarization of the membrane and an increase in Ca^2+^ influx, resulting in exocytosis of insulin granules and vice versa (Wei et al., 2019). Therefore, it seems that the closure of K_ATP_ channels by BCL can be a therapeutic target in the treatment of diabetes, and further studies should be conducted to evaluate the hypoglycemic effects of herbal medicine on K_ATP_ channels in the pancreas. A possible mechanism of the interaction of BCL with K^+^ channels in diabetes is shown in Figure 5.

### The Modulatory Effects of Medicinal Plants on NO/cGMP/K_ATP_ Cascade in the Gastrointestinal Tract

We found two studies related to the gastroprotective and antimotility effects of medicinal plants in the gastrointestinal tract, which summarize their possible molecular mechanisms.

#### The Involvement of NO/cGMP/K_ATP_ Pathway in Gastroprotective Effects

HECr shows gastroprotective effects through non-specific complexes, including activation of K_ATP_ channels, α_2_-adrenergic receptors, and stimulation of PGs and NO (Arunachalam et al., 2019). Recently, the efficacy of several medicinal plants and possible mechanisms for the treatment of peptic ulcer disease has been discussed (Ardalani et al., 2020). One of the possible gastroprotective effects of medicinal plants and their active constituents is via the NO/cGMP/K_ATP_ pathway (Serafim et al., 2020). Endothelial nitric oxide synthase releases NO. NO stimulates soluble guanylyl cyclase (sGC) and increases cGMP in smooth muscle cells, opening the K_ATP_ channels (da Silva Monteiro et al., 2019). The efflux of K^+^ blocks the voltage-sensitive calcium channels, which relaxes the smooth muscle, improves blood flow, and facilitates the healing process (Serafim et al., 2020). PGs are another mediator that could activate the K_ATP_ channels (Peskar et al., 2002).

**FIGURE 6:**
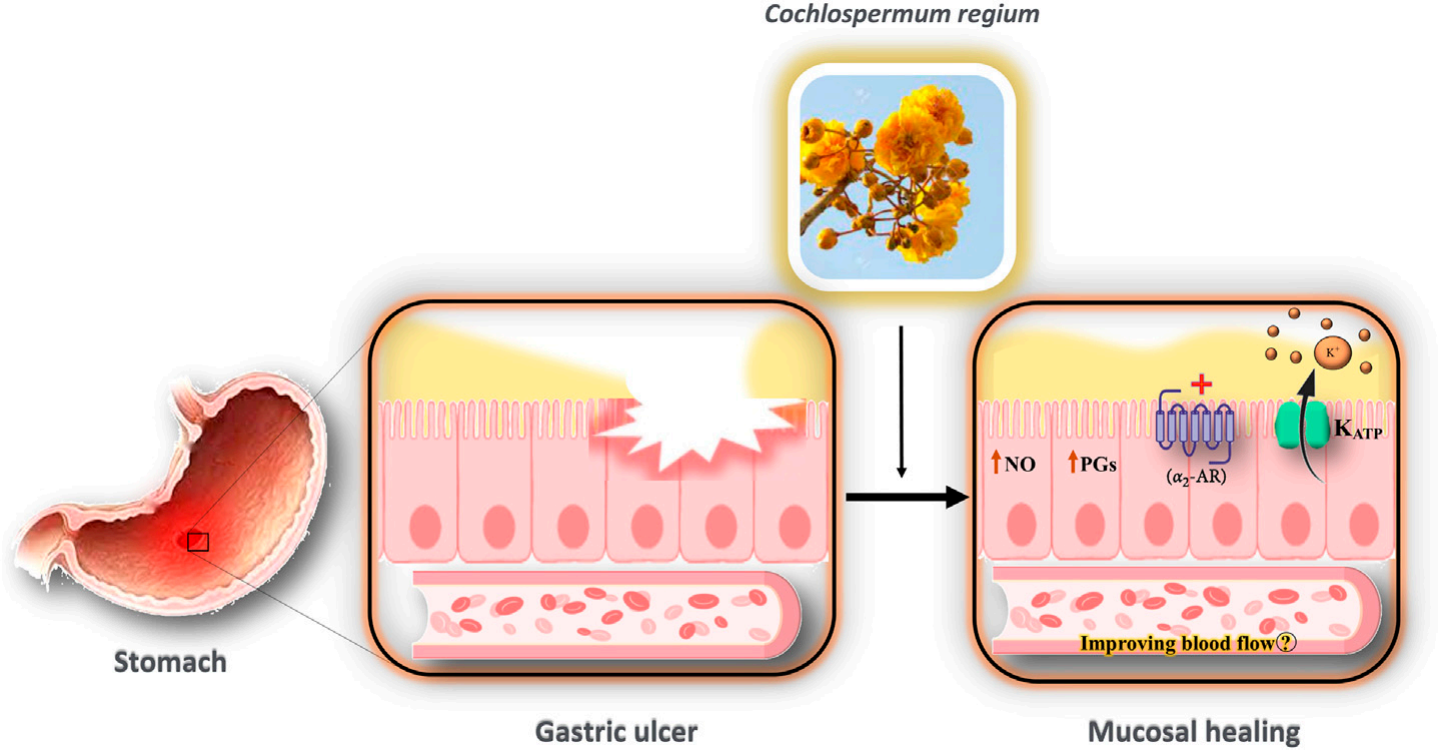
Possible mechanism of the interaction of HECr with K^+^ channels in the gastrointestinal tract. The gastroprotective effect of HECr occurs non-specifically through the activation of K^+^_ATP_ channels and α_2_-adrenergic receptors, and the stimulation of PGs and NO. The efflux of K^+^ blocks the voltage-sensitive calcium channels, which relaxes the smooth muscle, improves blood flow, and facilitates the healing process. NO, nitric oxide; PGs, prostaglandins; K^+^, potassium; K_ATP_, ATP-sensitive potassium channel; α_2_-AR, alpha-2 adrenergic receptor.

Drugs that could open K_ATP_ channels can protect against gastric and small intestine injury induced by ethanol or indomethacin in animals (Akar et al., 1999; Peskar et al., 2002; Menozzi et al., 2011). Since the activation of NO, K_ATP_, and PGs, apart from α_2_-adrenergic receptors, have gastroprotective effects (Figure 6), it seems that the modulation of the NO/cGMP/K_ATP_ signaling by medicinal plants should be considered as a novel therapeutic target for the treatment of gastrointestinal ulcers.

#### The Involvement of the NO/cGMP/K_ATP_ Pathway for Antimotility

Formiga et al. (2017) indicated that the NO/cGMP/K_ATP_ pathway is involved in the inhibitory effect of ME on intestinal motility in mice. However, the modulation of the adrenergic system by ME should not be overlooked (Formiga et al., 2017). NO is an inhibitory co-transmitter released from the enteric nervous system. NO activates sGC, leading to the production of cGMP, which in turn activates K^+^ channels (Matsuda and Miller, 2010; Modzelewska et al., 2021). The expression of subunits Kir6.2/SUR2B in K_ATP_ channels has been reported in the murine colon (Koh et al., 1998; Currò, 2016). It has been shown that the activation of K_ATP_ channels leads to resting membrane potential in the colonic smooth muscle of some species (Currò, 2016). The important functional roles of the inwardly rectifying type 6 K^+^ (Kir6) and K_V_1.2, 1.5, 2.2, 4.3, 7.4, and 11.1, and K_Ca_1.1 and 2.3 channels were determined in the gastrointestinal smooth muscle. Theoretically, activators of these channels may relax these muscles, thereby promising a new therapeutic target for functional gastrointestinal disorders (Currò, 2016). Hence, the activation of the NO/cGMP/K_ATP_ pathway by medicinal plants could be considered as a novel target for the treatment of motility disorders in the gastrointestinal tract.

### The Modulatory Effects of Medicinal Plants or Their Active Constituents on K^+^ Channels in the Cardiovascular System

We found two studies related to the cardioprotective and hypotensive effects of polydatin and ESAH.

#### The Involvement of PKC-K^+^_ATP_ Signaling for Antiischemic/Reperfusion Injury

**FIGURE 7:**
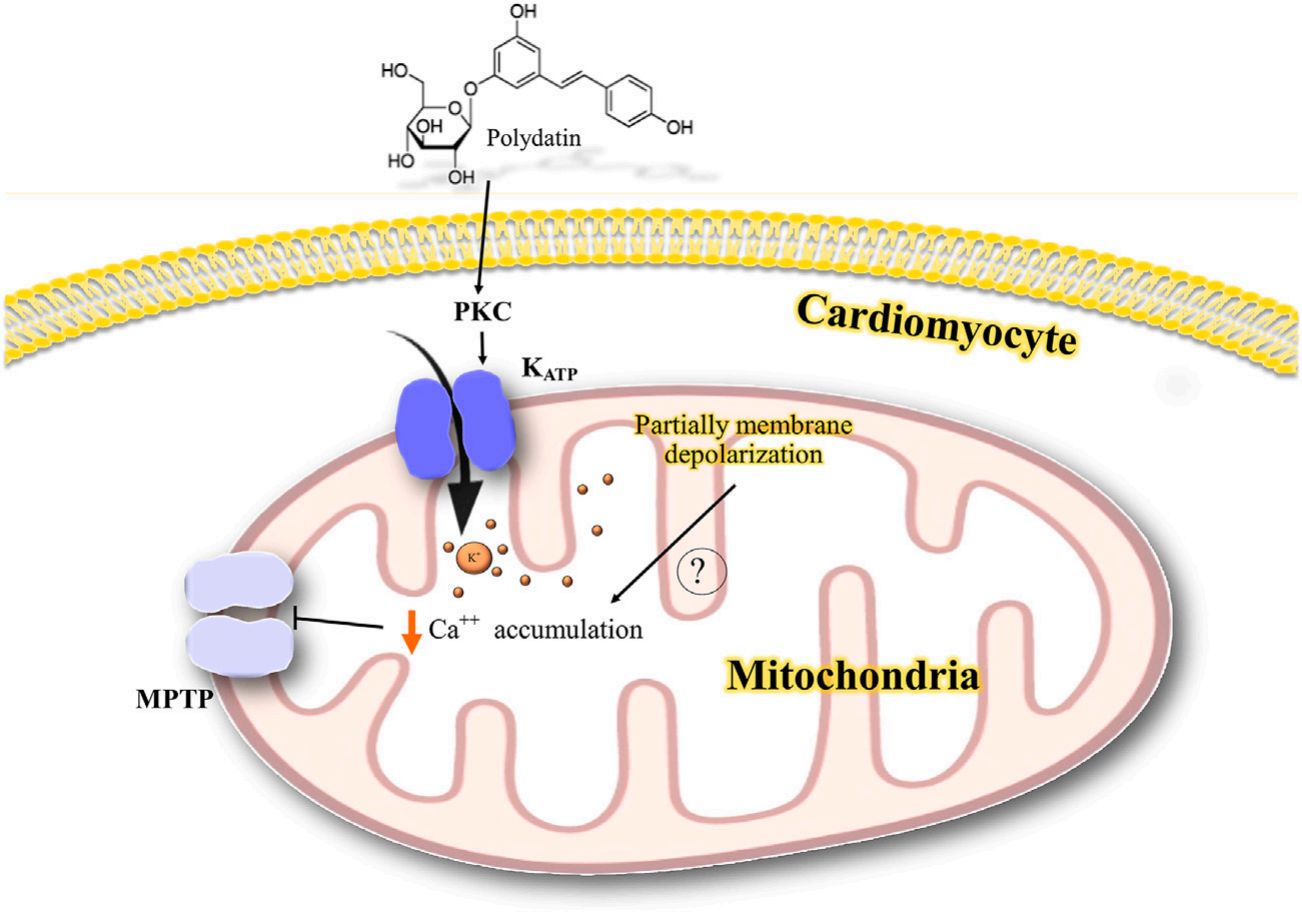
Possible mechanism of the interaction of polydatin with K^+^ channels in the cardiovascular system. PKC activates mitochondrial K_ATP_ channels possibly leading to a partial depolarization of mitochondrial potential and reducing the mitochondrial calcium accumulation and inhibiting mitochondrial permeability transition. PKC, protein kinase C; K^+^, potassium; Ca^++^, calcium; K_ATP_, ATP-sensitive potassium channel; MPTP, mitochondrial permeability transition pore.

The role of PKC-K^+^_ATP_ signaling was demonstrated by the finding that 5-HD and two PK inhibitors reversed the cardioprotective effect of polydatin in ischemia/reperfusion injury (Miao et al., 2011). The cardioprotective role of PKC has been reported in ischemic preconditioning (Liu et al., 1996; Murata et al., 2001; Perricone and Vander Heide, 2014; Foster and Coetzee, 2016). PKC could activate mitochondrial K_ATP_ channels, possibly leading to an increase in the resistance of mitochondria to mitochondrial permeability transition (Perricone and Vander Heide, 2014). It has been suggested that opening mitochondrial K_ATP_ channels partially depolarizes the mitochondrial potential, leading to attenuation of mitochondrial calcium accumulation in ischemia by decreasing the overload of Ca^2+^ (Murata et al., 2001). A possible mechanism for the interaction of polydatin with K^+^ channels in the cardiovascular system is shown in Figure 7.

#### Involvement of NO/cGMP/K^+^ Channels for Hypotensive Effects

Tirloni et al. (2017) showed that the hypotensive effect of ESAH is linked to the involvement of NO/cGMP and K^+^ channels in normotensive rats. The hypotensive effect of ESAH was completely inhibited by l-NAME or methylene blue. Thus, it seems that the NO/cGMP signaling also contributes to this effect. However, it is not known how this extract increased endothelial NO activity, which increased cGMP and, in turn, opened K^+^ channels. Therefore, the trigger for NO release needs to be investigated. However, it seems that the NO/cGMP/K channels could be a new target in the treatment of hypertension (Tirloni et al., 2017).

### Methodological Quality/Risk of Bias

The assessment of risk bias is shown in Table 3. Most studies scored 4–6 in our validation. All papers have a low risk of bias for the three domains of “incomplete outcome data,” “selective outcome reporting,” and “other sources of bias.” “Baseline characteristic” is observed with a low risk of bias in 11 publications (84.61%). “Random housing” was reported by six publications (46.15%). However, we found that one study (7.69%) had a low risk of bias in “blinding caregivers and/or investigators”. Furthermore, RoB was unclear for “random sequence generation,” “allocation concealment,” “random outcome assessment,” and “blinding outcome” for 13 papers.

**TABLE 3:** Assessment of risk of bias.

Study	1	2	3	4	5	6	7	8	9	10	Score
Adeyemi et al. (2018)	?	+	?	-	?	?	?	+	+	+	4
Brandão et al. (2013)	?	+	?	-	?	?	?	+	+	+	4
Ferdous et al. (2020)	?	+	?	-	?	?	?	+	+	+	4
Islam et al. (2016)	?	+	?	+	?	?	?	+	+	+	5
Khalid et al. (2011)	?	+	?	+	+	?	?	+	+	+	6
Shajib et al. (2018)	?	+	?	+	?	?	?	+	+	+	5
Han et al. (2018)	?	+	?	+	?	?	?	+	+	+	5
Adongo et al. (2017)	?	+	?	-	?	?	?	+	+	+	4
Wu et al. (2011)	?	-	?	+	?	?	?	+	+	+	4
Arunachalam et al. (2019)	?	+	?	-	?	?	?	+	+	+	4
Formiga et al. (2017)	?	+	?	-	?	?	?	+	+	+	4
Miao et al. (2011)	?	-	?	+	?	?	?	+	+	+	4
Tirloni et al. (2017)	?	+	?	-	?	?	?	+	+	+	4

## Limitations

This study represents the first systematic evaluation of preclinical *in vivo* studies related to the effects of medicinal plants or phytochemicals on different K^+^ channels. Among the screened databases, only 13 studies met the inclusion criteria. We excluded all *in vitro* or both *in vivo* and *in vitro* studies to measure the RoB as the guideline of SYRCLE for animal intervention studies. Another limitation is the lack of clinical studies on the therapeutic role of herbal medicines in K^+^ channel-related diseases. Furthermore, the interaction of herbal medicines and their active constituents with several K^+^ channels was determined in various organs with different functions. Hence, a meta-analysis is not feasible because of the heterogeneity among these studies.

## Conclusion

Dysregulation of K^+^ channels has been implicated in the pathophysiology of cardiovascular, gastrointestinal, neurological, and metabolic disorders. This is the first systematic review to show the various biological effects of medicinal plants and their constituents on hypotensive, antiischemic, antidiarrheal, antispasmodic, anti-inflammatory, antinociceptive, and hypoglycemic effects. These effects have been linked to the modulation of the activity of K_ATP_, SK_Ca_, BK_Ca,_ and K^+^ channels via possible involvement of the NO/cGMP pathway and PKC. Hence, K^+^ channels should be considered as significant therapeutic milestones in the treatment of several diseases. Future studies should focus on new technologies to study phytochemicals or their active constituents that interact with K^+^ channels to develop novel antinociceptive, anticonvulsant, cardioprotective, gastroprotective, and anti-ischemic therapeutics. We believe that this review will be a reliable guide for the target development and drug design for K^+^ channel-related disorders.

## Data Availability

The raw data supporting the conclusions of this article will be made available by the authors, without undue reservation.
